# Fangcang shelter hospitals during the COVID-19 epidemic, Wuhan, China

**DOI:** 10.2471/BLT.20.258152

**Published:** 2020-09-29

**Authors:** Juan Li, Pei Yuan, Jane Heffernan, Tingting Zheng, Nick Ogden, Beate Sander, Jun Li, Qi Li, Jacques Bélair, Jude Dzevela Kong, Elena Aruffo, Yi Tan, Zhen Jin, Yong Yu, Meng Fan, Jingan Cui, Zhidong Teng, Huaiping Zhu

**Affiliations:** a Centre for Disease Modelling, York University, 4700 Keele Street, Toronto, Ontario, M3J 1P3, Canada.; b College of Mathematics and System Science, Xinjiang University, Urumqi, China.; c Public Health Risk Sciences Division, Public Health Agency of Canada, Canada.; d Toronto Health Economics and Technology Assessment Collaborative, University Health Network, Toronto, Canada.; e School of Mathematics and Statistics, Xidian University, Xi’an, China.; f Department of Mathematics, Shanghai Normal University, Shanghai, China.; g Département de Mathématiques et de Statistique, Université de Montréal, Montréal, Canada.; h Complex System Research Center, Shanxi University, Taiyuan, China.; i School of Public Health and Management, Hubei University of Medicine, Shiyan, China.; j School of Mathematics and Statistics, Northeast Normal University, Changchun, China.; k Department of Mathematics, Beijing University of Civil Engineering and Architecture, Beijing, China.

## Abstract

**Objective:**

To design models of the spread of coronavirus disease-2019 (COVID-19) in Wuhan and the effect of Fangcang shelter hospitals (rapidly-built temporary hospitals) on the control of the epidemic.

**Methods:**

We used data on daily reported confirmed cases of COVID-19, recovered cases and deaths from the official website of the Wuhan Municipal Health Commission to build compartmental models for three phases of the COVID-19 epidemic. We incorporated the hospital-bed capacity of both designated and Fangcang shelter hospitals. We used the models to assess the success of the strategy adopted in Wuhan to control the COVID-19 epidemic.

**Findings:**

Based on the 13 348 Fangcang shelter hospitals beds used in practice, our models show that if the Fangcang shelter hospitals had been opened on 6 February (a day after their actual opening), the total number of COVID-19 cases would have reached 7 413 798 (instead of 50 844) with 1 396 017 deaths (instead of 5003), and the epidemic would have lasted for 179 days (instead of 71).

**Conclusion:**

While the designated hospitals saved lives of patients with severe COVID-19, it was the increased hospital-bed capacity of the large number of Fangcang shelter hospitals that helped slow and eventually stop the COVID-19 epidemic in Wuhan. Given the current global pandemic of COVID-19, our study suggests that increasing hospital-bed capacity, especially through temporary hospitals such as Fangcang shelter hospitals, to isolate groups of people with mild symptoms within an affected region could help curb and eventually stop COVID-19 outbreaks in communities where effective household isolation is not possible.

## Résumé

**Objectif:**

Concevoir des modèles de propagation de la maladie à coronavirus 2019 (COVID-19) à Wuhan et mesurer l'impact des hôpitaux de campagne de type Fangcang (hôpitaux provisoires construits rapidement) sur le contrôle de l'épidémie.

**Méthodes:**

Nous avons utilisé les données relatives au nombre quotidien de cas confirmés de COVID-19, de guérisons et de décès publiées sur le site officiel de la Commission municipale de la santé de Wuhan pour établir des modèles compartimentaux correspondant aux trois phases de l'épidémie de COVID-19. Nous avons également tenu compte de la capacité d'accueil des hôpitaux de référence ainsi que des hôpitaux de campagne de type Fangcang. Nous avons employé ces modèles afin d’évaluer le succès de la stratégie adoptée à Wuhan pour endiguer l'épidémie de COVID-19.

**Résultats:**

En se basant sur les 13 348 lits réellement occupés dans les hôpitaux de campagne de type Fangcang, nos modèles ont montré que si ces structures temporaires avaient ouvert leurs portes le 6 février (un jour après leur ouverture effective), le nombre total de cas de COVID-19 aurait atteint 7 413 798 (au lieu de 50 844), entraînant 1 396 017 décès (au lieu de 5003), et l'épidémie aurait duré 179 jours (au lieu de 71).

**Conclusion:**

Bien que les hôpitaux de référence aient permis de sauver la vie des patients souffrant d'une forme grave de COVID-19, c'est surtout l'augmentation de la capacité d'accueil des multiples hôpitaux de campagne de type Fangcang qui a contribué à ralentir, puis à enrayer l'épidémie de COVID-19 à Wuhan. Dans le cadre de l'actuelle pandémie de COVID-19, notre étude suggère qu'un accroissement de la capacité d'accueil des hôpitaux, notamment par le biais de structures temporaires de type Fangcang, afin d'isoler les groupes présentant peu de symptômes au sein d'une région touchée pourrait limiter la propagation et les flambées épidémiques de COVID-19 dans les communautés où il est impossible d'instaurer un isolement efficace à domicile.

## Resumen

**Objetivo:**

Diseñar modelos sobre la propagación de la enfermedad por coronavirus de 2019 (COVID-19) en Wuhan y sobre el impacto de los hospitales de confinamiento Fangcang (hospitales temporales construidos con rapidez) en el control de la epidemia.

**Métodos:**

Se usaron los datos sobre los casos confirmados y notificados diariamente de la COVID-19, los casos recuperados y las muertes del sitio web oficial de la Comisión Municipal de Salud de Wuhan para elaborar modelos compartimentales de las tres fases de la epidemia de la COVID-19. Se integró la capacidad de las camas de los hospitales designados y de confinamiento Fangcang. Se aplicaron los modelos para evaluar el éxito de la estrategia que se adoptó en Wuhan para controlar la epidemia de la COVID-19.

**Resultados:**

Teniendo en cuenta las 13 348 camas de los hospitales de confinamiento Fangcang que se emplearon en la práctica, los modelos indican que si los hospitales de confinamiento Fangcang se hubieran abierto el 6 de febrero (un día después de su apertura efectiva), el número total de los casos de la COVID-19 habría alcanzado los 7 413 798 (en lugar de 50 844) y se habrían producido 1 396 017 muertes (en lugar de 5003), por lo que la epidemia habría durado 179 días (en lugar de 71).

**Conclusión:**

Si bien los hospitales designados salvaron vidas de pacientes que padecían la COVID-19 grave, fue el aumento de la capacidad de las camas en los hospitales de confinamiento Fangcang lo que ayudó a frenar y finalmente detener la epidemia de la COVID-19 en Wuhan. Dada la actual pandemia mundial de la COVID-19, el presente estudio sugiere que el aumento de la capacidad de las camas en los hospitales, en especial en los hospitales temporales como los hospitales de confinamiento Fangcang, para aislar a los grupos de personas con síntomas leves dentro de una región afectada podría ayudar a frenar y finalmente detener los brotes de la COVID-19 en las comunidades donde el aislamiento doméstico eficaz no es posible.

## ملخص

الغرض تصميم نماذج لانتشار مرض فيروس كورونا عام 2019 (كوفيد 19) في ووهان، وتأثير مستشفيات العزل في فانجكانج (مستشفيات مؤقتة يتم بناؤها بسرعة) في السيطرة على الوباء.

الطريقة قم باستخدام بيانات عن الحالات المؤكدة المبلغ عنها يوميًا لكوفيد 19، والحالات المتعافية والوفيات من الموقع الإلكتروني الرسمي للجنة الصحة في بلدية ووهان لبناء نماذج مجزأة لثلاث مراحل من جائحة كوفيد 19. قمنا بدمج سعة الأسرّة بالمستشفى لكل من المستشفيات المخصصة ومستشفيات العزل في فانجكانج. استخدمنا النماذج لتقييم نجاح الاستراتيجية المعتمدة في ووهان للسيطرة على جائحة كوفيد 19.

النتائج اعتمادًا على عدد 13348 سريرًا في مستشفيات العزل بفانجكانج المستخدمة في العلاج، تُظهر نماذجنا أنه إذا ما تم فتح مستشفيات العزل في فانجكانج في 6 فبراير/شباط (بعد يوم من الافتتاح الفعلي)، لكان إجمالي عدد الحالات المصابة بكوفيد 19 قد وصل إلى 7 413 798 (بدلاً من 50844) مع 1 396 017 حالة وفاة (بدلاً من 5003)، وكانت الجائحة ستستمر لمدة 179 يومًا (بدلاً من 71).

الاستنتاج بينما أنقذت المستشفيات المعينة حياة المرضى المصابين بحالة حادة من مرض كوفيد 19، كانت زيادة سعة الأسرّة بالمستشفيات لعدد كبير من مستشفيات العزل في فانجكانج، هي التي ساعدت في إبطاء وتيرة انتشار جائحة كوفيد 19، وإيقافها في النهاية في ووهان. مع وضع الجائحة العالمية الحالية لكوفيد 19 في الاعتبار، فإن دراستنا تشير إلى أن زيادة سعة الأسرّة بالمستشفيات، وخاصة من خلال المستشفيات المؤقتة مثل مستشفيات العزل في فانجكانج، لعزل مجموعات الأشخاص المصابين بأعراض خفيفة داخل منطقة متضررة، يمكن أن يساعد في الحد من تفشي جائحة كوفيد 19، ووقفها في النهاية، في المجتمعات التي لم يمكن العزل المنزلي الفعال فيها ممكناً.

## 摘要

**目的:**

设计武汉 2019 年冠状病毒 (COVID-19) 传播模型，探究方舱庇护医院（快速建成的临时医院）对控制疫情的效果。

**方法:**

我们利用武汉市卫健委官网每日发布的 COVID-19 确诊病例、治愈病例以及死亡病例数据，构建 COVID-19 疫情三阶段的房室模型。我们整合了定点医院和方舱庇护医院的床位数。利用这些模型评估武汉控制 COVID-19 疫情时采取的战略是否成功。

**结果:**

依据方舱庇护医院实际投入使用 13348 张床位，我们的模型显示，如果其于 2 月 6 日（实际开放后第二天）开放，新冠病毒肺炎 (COVID-19) 确诊病例总数则会高达 7 413 798（而非 50844），死亡病例则会高达 1 396 017（而非 5003），且疫情将会持续 179 天（而非 71 天）。

**结论:**

定点医院救治了新冠病毒肺炎 (COVID-19) 重症患者，同时也正是大量方舱庇护医院增加床位，才得以降低扩散速度，从而最终遏制住武汉新冠病毒肺炎 (COVID-19) 疫情。鉴于当前新冠病毒肺炎 (COVID-19) 全球大流行，我们的研究表明增加床位，尤其通过建造方舱庇护医院这类临时医院，隔离疫区轻度患者，有助于无法实施有效家庭隔离的社区控制疫情，最终结束新冠病毒肺炎 (COVID-19) 疫情。

## Резюме

**Цель:**

Разработать модели распространения коронавирусного заболевания 2019 г. (COVID-19) в г. Ухань и влияния больниц-приютов поселения Фангкан (быстро отстроенные временные больницы) на борьбу с эпидемией.

**Методы:**

Авторы использовали данные о ежедневно регистрируемых подтвержденных случаях COVID-19, случаях выздоровления и смертях с официального сайта Муниципальной комиссии по здравоохранению г. Ухань, чтобы построить компартментные модели для трех фаз эпидемии COVID-19. Также авторы включили число коек в назначенных больницах и в больницах-приютах поселения Фангкан. Модели были использованы для оценки успеха стратегии, принятой в г. Ухань для борьбы с эпидемией COVID-19.

**Результаты:**

Основываясь на фактически использованном количестве коек (13 348 коек) в больницах-приютах поселения Фангкан, наши модели показывают, что, если бы больницы-приюты Фангкан были открыты 6 февраля (на следующий день после их фактического открытия), общее количество случаев COVID-19 достигло бы 7 413 798 человек (вместо 50 844), при этом количество смертей составило бы 1 396 017 (вместо 5003), а эпидемия продолжалась бы 179 дней (вместо 71).

**Вывод:**

В то время как назначенные больницы спасали жизни пациентов с тяжелой формой COVID-19, именно увеличение количества коек в большинстве больниц-приютов поселения Фангкан помогло замедлить и в конечном итоге остановить эпидемию COVID-19 в г. Ухань. С учетом текущей глобальной пандемии COVID-19 наше исследование указывает на то, что увеличение числа больничных коек, особенно за счет временных больниц, таких как больницы-приюты Фангкан, для изоляции групп людей со слабо выраженными симптомами в пострадавшем регионе способно помочь сократить и в конечном итоге остановить возникновение вспышек COVID-19 в сообществах, в которых невозможно обеспечить эффективную домашнюю изоляцию.

## Introduction

On 30 January 2020, the World Health Organization declared coronavirus disease-2019 (COVID-19) a public health emergency of international concern.^1^ The disease was first reported in Wuhan, China, in December 2019. To control the COVID-19 epidemic, Wuhan, a city with an estimated population of 10 million, started a lockdown on 23 January 2020.^2^ The city itself was quarantined and turned into an isolation ward.^3^

To alleviate the shortage of doctors and medical resources, medical teams and materials were dispatched in batches to Wuhan from other parts of China.^4^ Several hospitals in Wuhan were designated as COVID-19 hospitals and their capacity to accept daily confirmed cases of COVID-19 was increased.^5^^,^^6^ However, the number of confirmed cases continued to grow even though quarantine and social-distancing policies were strictly enforced.^7^ The situation with the epidemic did not improve until the opening of Fangcang shelter hospitals on 5 February 2020. Fangcang shelter hospitals are rapidly built temporary hospitals composed of several movable shelters; they are equipped to provide services such as emergency treatment, surgical treatment and clinical examination.^8^

To effectively control the spread of COVID-19, the government of Wuhan decided to move all COVID-19 patients together, enlist all experts in infectious diseases and doctors (health-care personnel) and centralize all resources.^9^^,^^10^ To implement these policies, on 3 February, the decision was taken to treat patients by severity of infection and to start building Fangcang shelter hospitals for mild cases, who did not need intensive care. This approach effectively changed the family-based quarantine approach into group isolation of mild confirmed cases.^11^

**Fig. 1:**
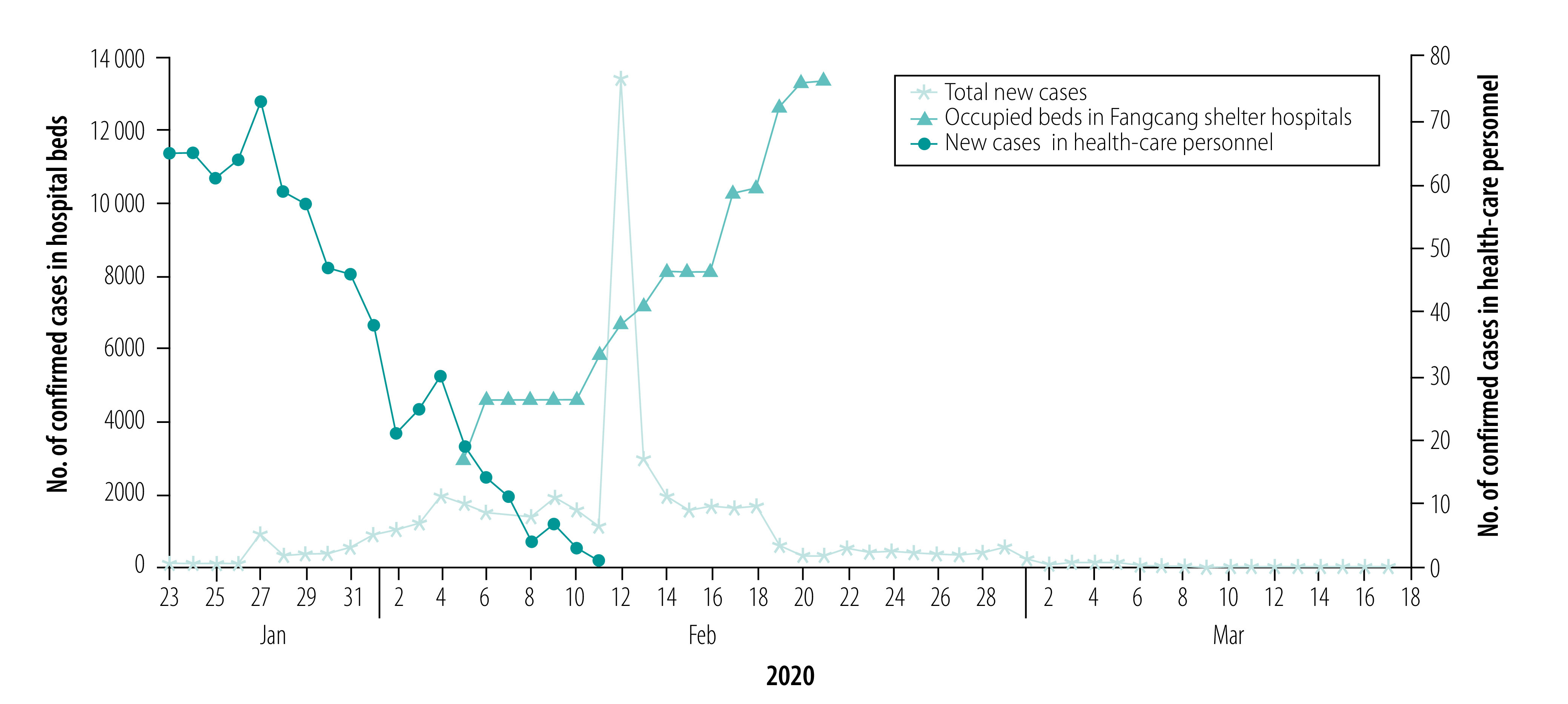
Daily number of confirmed cases of COVID-19 in total (23 January–17 March) and in health-care personnel (23 January–11 February), and cumulative daily number of occupied hospital beds in Fangcang shelter hospitals (5–22 February), Wuhan, China, 2020

Wuhan continued to build more Fangcang shelter hospitals and by the middle of February, the daily number of new confirmed cases started to decrease (Fig. 1). While the availability of hospital beds in both designated hospitals and Fangcang shelter hospitals and sufficient health-care personnel were important in minimizing the transmission of severe acute respiratory syndrome coronavirus 2 (SARS-CoV-2) in the city, it was recognized that front-line health-care personnel in close contact with infectious patients had a much higher risk of infection. By 11 February, 3019 health-care personnel had been infected and five had died.^13^

COVID-19: coronavirus disease-2019.

Data source: Wuhan Municipal Health Commission.^12^

Here, we build compartmental models to mimic the spread of COVID-19 in Wuhan and examine how the lockdown policy controlled the epidemic. We computed the basic reproduction numbers to assess the risk of infection in health-care personnel and the general public. We also estimated the number of hospital beds needed to control the COVID-19 epidemic. Many modelling studies of COVID-19 in Wuhan have been done;^14^^–^^17^ however, these models did not consider the roles played by the beds in the Fangcang shelter hospitals and health-care personnel in the infection dynamic.

## Methods

### Data used

The epidemic of COVID-19 in Wuhan resulted in 50 003 infections and 2469 deaths as of 15 March 2020.^18^ We obtained data on the reported daily new confirmed cases of COVID-19, recovered cases and deaths from 23 January to 17 March from the official website of the Wuhan Municipal Health Commission.^12^ When lockdown started, testing resources and health-care personnel were limited. As a result, the data are affected by a testing time lag from the date of onset of symptoms to the date of the test result and, therefore, the number of confirmed cases reported in the data are not the actual number of infections on a specific day. In addition, the diagnostic criteria for COVID-19 were updated five times. As shown in Fig. 1, the daily number of confirmed cases increased substantially on 13 February, reaching about 12 000. This sudden jump can be attributed to a change in national test standards.^19^ Hence, we used data only from 12 February onwards to estimate the model parameters and initial variables.

In our models, we treated health-care personnel separately from the general public. We extracted data on the cumulative number of infected health-care personnel in Wuhan, Hubei province (excluding Wuhan) and daily infected health-care personnel in the whole country^13^^,^^20^^,^^21^ to calculate the daily infection of health-care personnel in Wuhan from 23 January to 11 February. When the lockdown started, many health-care personnel were infected because of the limited hospital capacity to handle the large number of patients (Fig. 1). The number of reported cases among health-care personnel started to decrease in the middle of February when a large number of hospital beds were added through the building of the Fangcang shelter hospitals. As an indication of the effectiveness of control measures, we estimated the bed capacity of the designated hospitals and Fangcang shelter hospitals from data in public reports for Wuhan.^13^^,^^22^ We denoted *T*_0_ as the date when the lockdown started (23 January 2020), *T*_1_ as the date when the first bed in the Fangcang shelter hospitals opened (5 February) and *T*_2_ as the date when no new Fangcang shelter hospital beds were installed and more of the beds became free (22 February). We defined phase I of the epidemic as the period from *T*_0_ to *T*_1_, phase II as the period from *T*_1_ to *T*_2_, and phase III as the period from *T*_2_ to the time that our simulations were terminated. We denoted *B*_1_(*t*) as the total number of designated-hospital beds. Thus, if *b*_1_(*t*) is the number of new beds for COVID-19 patients installed a day in the designated hospitals, then
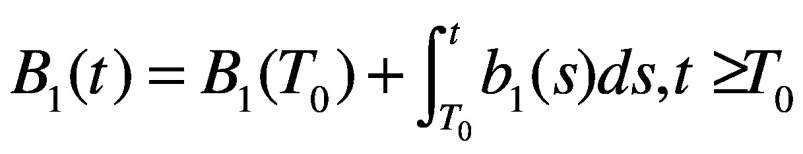
(1)Similarly, we defined the total number of beds in Fangcang shelter hospitals as:
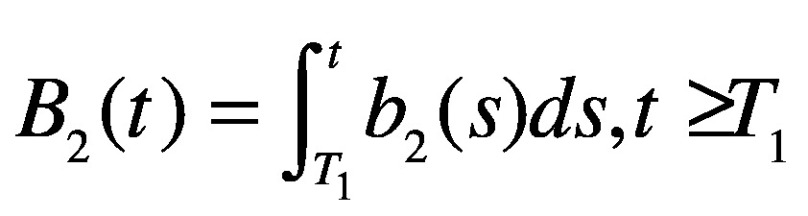
(2)where *b*_2_(*t*) is the number of new beds installed per day in Fangcang shelter hospitals.

In a standard designated hospital or Fangcang shelter hospital, given the limited resources, the ratio of number of beds to number of health-care personnel is usually fixed to ensure proper care. We use *k*_1_ and *k*_2_ to denote these ratios in designated hospitals and Fangcang shelter hospitals, respectively. With information from the Wuhan Municipal Health Commission^22^ and news report,^23^^,^^24^ we calculated *k*_1_ as 2.486 and *k*_2_ as 1.107, from which we determined the total number of health-care personnel. With the construction and assignment of designated and Fangcang shelter hospitals, the number of hospital beds used became an important quantity to reflect the progression and severity of the epidemic. We defined *V*_1_(*t*) and *V*_2_(*t*) as the number of available beds in designated hospitals and Fangcang shelter hospitals, respectively. The number of beds started to increase on 15 February and the cumulative number of new confirmed cases started to decrease on 22 February. Therefore, we did not count the extra beds installed after 22 February, even though the plan to build more Fangcang shelter hospitals continued. We thus determined *B*_2_(22) as 13 348, the limit of *B*_2_(*t*). In addition, we defined the end date of the COVID-19 epidemic in Wuhan to coincide with the date that satisfies *B_i_*(*t*) *= V_i_(t), I =* 1,2, when empty beds in the designated hospitals and Fangcang shelter hospitals are available to admit new patients. Note that the daily number of newly built beds planned was not the actual number opened and all beds were opened on a day to day basis as needed.^25^^–^^27^ We used the moving average method to smooth the cumulative number of beds to calculate the daily number of new beds put into use (available in the data repository).^28^

### Models

We developed deterministic susceptible-exposed-asymptomatic-infectious-recovered models for the three phases of the epidemic based on the assumptions listed in Box 1 (available at: http://www.who.int/bulletin/volumes/98/12/20-258152). We divided the population into three groups: non-health-care personnel (subscript *w*), health-care personnel in designated hospitals (subscript *h*) and health-care personnel in Fangcang shelter hospitals (subscript *g*). We further classified these groups as: susceptible (*S_i_*(*t*)), exposed (*E_i_*(*t*)), asymptomatic (subclinical) infection (*A_i_*(*t*)), infectious pre-symptomatic (will eventually show symptoms) (*I_i_*_1_(*t*)), infectious symptomatic (*I_i2_*(*t*)) and recovered (*R_i_*(*t*)). Based on the flowcharts for the three phases of the epidemic (data repository),^28^ we established model equations accordingly (data repository).^28^

Box 1 **Assumptions used to model the COVID-19 epidemic, Wuhan, China, 2020**

1. Natural births and deaths are not considered. No population movement.

2. All susceptible individuals exposed to the virus have the same probability of infection.

3. Subclinical infected cases will recover from the infection and will not be reinfected.^29^

4. Both *A_i_*(*t*) and *I_i1_*(*t*) are infectious virus carriers. Individuals in *A_i_*(*t*) will never show symptoms, while individuals in *I_i1_*(t) will develop into symptomatic cases (*I_i2_*(*t*)) after a specified period of time.

5. Infected health-care personnel who are asymptomatic will continue to work in the hospitals.

6. Transmission between members of the public who are not admitted to hospital and health-care personnel is not considered.

7. The epidemic course in Wuhan had three phases: phase I from *T*_0_ (23 January) to *T*_1_ (5 February), phase II from *T*_1_ (5 February) to *T*_2_ (22 February) and phase III after *T*_2_ (22 February). In phase I, only designated hospitals were operating. Fangcang shelter hospitals were built to increase bed capacity starting in phase II. In phase III, all patients with mild and severe confirmed COVID-19 could be admitted immediately to the designated hospitals and Fangcang shelter hospitals. *I_w2_* will not be considered as a single variable in phase III any longer.

8. Health-care personnel with symptoms of COVID-19 will be admitted and given priority for hospital beds. In phase I, health-care personnel confirmed with COVID-19 will be admitted to the designated hospitals. After phase I, health-care personnel with mild symptoms will be admitted to Fangcang shelter hospitals with priority.

9. Non-health-care personnel are admitted into Fangcang shelter hospitals or designated hospitals depending on their symptoms (mild or severe) and on the number of beds that will be used for health-care personnel in Fangcang shelter hospitals and designated hospitals.

10. Severely infected individuals are admitted to designated hospitals and enter the *I_wB1_*(*t*) class, after which they recover to *R_h_*(*t*) (the class of all those recovered from the infection at the designated hospitals), or die.

11. From phase II, COVID-19 cases with mild disease are admitted to the Fangcang shelter hospitals as a class we denote as I*_wB2_*(t). This class can recover to *R_g_*(*t*) or can be admitted to designated hospitals if they develop severe symptoms and enter the *I_wB1_*(*t*) class.

12. Patients in designated hospitals whose condition is improving will not be moved to Fangcang shelter hospitals.

COVID-19: coronavirus disease-2019.

We estimated model parameters and calculated the basic reproduction number *R*_0_ for phase III using the next-generation matrix method.^30^ Using *R*_0_, we defined the instantaneous risk index as *R*_0_(*t*).^30^^,^^31^ The formulas for *R*_0_ and *R*_0_(*t*) are given in the data repository.^28^

### Sensitivity analysis

Given the uncertainty of the model parameters, we did a sensitivity analysis of key parameters, including the transmission rates (*βs*), the proportion of subclinical infections (*a*) and the number of beds in the designated hospitals and Fangcang shelter hospitals (*b*_1_,*b*_2_). We used the Latin hypercube sampling and partial rank correlation coefficient method.^32^ To examine how these parameters affected the transmission over the three phases of the epidemic, we generated 3000 samples of these parameters, using Latin hypercube sampling and varied them between 80% and 120% of their estimated values. We then verified the monotonic relationships between the parameters and the outcomes of the models. We calculated the values of the partial rank correlation coefficient, which determine the significance (partial rank correlation coefficient magnitude > 0.5 required) of each parameter to variations in the model outcomes.

### Simulations

We set the initial values and some parameters for each phase using the data available. On day*T_0_*, we set the initial values for variables for Fangcang shelter hospitals to zero (Table 1; available at: http://www.who.int/bulletin/volumes/98/12/20-258152). We estimated the initial values for the six state variables for non-health-care personnel and the 14 parameters associated with transmission using Bayesian methods. We assumed prior distributions of the parameters were multivariate Gaussian. We determined the values of the parameters as the mean of the posterior distributions, which we obtained using Markov chain Monte Carlo methods and used the adaptive Metropolis–Hastings algorithm with 150 000 iterations and a 90 000 iteration burn-in reference.^33^ We assessed chain convergence by the Geweke statistic with values greater than 0.9 indicating a satisfactory chain convergence.

**Table 1:** Variables used in the modelling and their initial values, Wuhan, China, 2020

Variable	Description	Initial value (95% CI^a^)	Source of values
*S_w_*(*t*)	Number of susceptible people who are not health-care personnel	11 060 000 (11 060 000–11 060 000)	Markov chain Monte Carlo
*E_w_*(*t*)	Number of exposed people who are not health-care personnel	6667.5 (6600.7–6750.9)	Markov chain Monte Carlo
*A_w_*(*t*)	Number of asymptomatic people with COVID-19 (who will never develop symptoms) who are not health-care personnel	13.758 (13.589–14.002)	Markov chain Monte Carlo
*I_w1_*(*t*)	Number of asymptomatic people with COVID-19 (who will develop symptoms) who are not health-care personnel	2956.4 (2944.8–2967.5)	Markov chain Monte Carlo
*I_w2_*(*t*)	Number of untreated symptomatic people with COVID-19 who are not health-care personnel	115.01 (113.92–115.83)	Markov chain Monte Carlo
*R_w_*(*t*)	Number of people who have recovered without entering the hospital who are not health-care personnel	1.5833 (1.5664–1.6033)	Markov chain Monte Carlo
*S_h_*(*t*)	Number of susceptible health-care personnel who work in designated hospitals	6692	Calculated^b^
*E_h_*(*t*)	Number of exposed health-care personnel who work in designated hospitals	426	Calculated^c^
*A_h_*(*t*)	Number of asymptomatic health-care personnel with COVID-19 (who will never develop symptoms) who work in designated hospitals	2	Wuhan Municipal Health Commission^18^
*I_h1_*(*t*)	Number of asymptomatic health-care personnel with COVID-19 (who will develop symptoms) who work in designated hospitals	190	Calculated^d^
*R_h_*(*t*)	Number of recovered patients from designated hospitals^e^	31	Wuhan Municipal Health Commission^12^
*I_wB1_*(*t*)	Total number of patients in designated hospitals^e^	2692	Wuhan Municipal Health Commission^12^
*S_g_*(*t*)	Number of susceptible health-care personnel who work in Fangcang shelter hospitals	0	NA
*E_g_*(*t*)	Number of exposed health-care personnel who work in Fangcang shelter hospitals	0	NA
*A_g_*(*t*)	Number of asymptomatic health-care personnel with COVID-19 (who will never develop symptoms) who work in Fangcang shelter hospitals	0	NA
*I_g1_*(*t*)	Number of asymptomatic health-care personnel with COVID-19 (who will develop symptoms) who work in Fangcang shelter hospitals	0	NA
*R_g_*(*t*)	Number of recovered patients from Fangcang shelter hospitals^e^	0	NA
*I_wB2_*(*t*)	Total number of patients in Fangcang shelter hospitals^e^	0	NA

CI: confidence interval; COVID-19: coronavirus disease-2019; NA: not applicable.

^a^ 95% highest posterior density interval.

^b^ Calculated by multiplying the initial number of beds in designated hospitals by *k*_1_ (ratio of beds to health-care personnel) = 2.486.

^c^ Calculated by summing the number of health-care personnel with COVID-19 in the first 7 days (assumed incubation period).

^d^ Calculated by summing the number of health-care personnel with COVID-19 in the first 3 days (assumed time to progress from infectious to symptomatic).

^e^ All patients including health-care personnel.

To further assess the effect of medical resources in controlling the COVID-19 epidemic in Wuhan, we calculated the hospital beds per 1000-infected-person ratios^34^ to quantify the minimum number of beds required in different scenarios. In the case of sufficient or increasing medical resources, we estimated the hospital beds per 1000-infected-person ratio as:
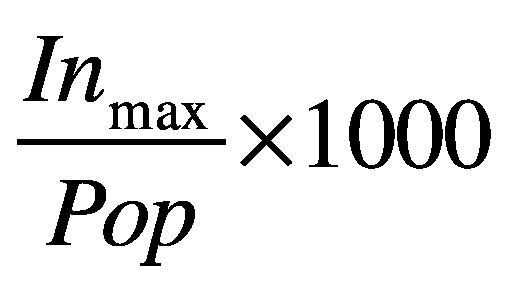
(3)where *Pop* is the total population in Wuhan and the maximum number of inpatients daily (*In_max_*) is determined by *I_wB1_(t*) + *I_wB2_(t)*. Larger values of the hospital beds per 1000-infected-person ratio mean more beds are needed to mitigate the epidemic.

## Results

Using the daily numbers of new beds in the designated hospital (23 January–25 February) and the Fangcang shelter hospitals (5–22 February), we fitted our model to the data of cumulative confirmed cases, recovered cases and deaths from 23 January to 17 March, and the cumulative number of cases among health-care personnel from 23 January to 11 February. The estimated parameters and highest density intervals are shown in Table 1 and Table 2 (available at: http://www.who.int/bulletin/volumes/98/12/20-258152).

**Table 2:** Parameter estimation for COVID-2019 in Wuhan, China

Parameter	Description	Estimated mean values (95% CI^a^)	Source of values
*β_w1_*	Infection rate of susceptible people (non-health-care personnel) by asymptomatic infectious individuals (non-health-care personnel)	3.3775 × 10^−10^ (3.3391 × 10^−10^–3.4015 × 10^−10^)	Markov chain Monte Carlo
*β_w2_*	Infection rate of susceptible people (non-health-care personnel) outside the designated hospitals or Fangcang shelter hospitals by infectious symptomatic individuals (non-health-care personnel)	5.4667 × 10^−8^ (5.4304 × 10^−8^–5.5022 × 10^−8^)	Markov chain Monte Carlo
*β_h_*^b^	Infection rate of susceptible health-care personnel in designated hospitals by infectious patients	Phase I: 1.2477 × 10^−7^ (1.2425 × 10^−7^–1.2527 × 10^−7^) Phase II–III: 1.6699 × 10^−9^ (1.6570 × 10^−9^–1.6814 × 10^−9^)	Markov chain Monte Carlo
*β_h1_*^c^	Infection rate of susceptible health-care personnel in designated hospitals by infectious asymptomatic health-care personnel in designated hospital	Phase I: 7.0175 × 10^−9^ (6.8941 × 10^−9^–7.1492 × 10^−9^) Phase II–III: 9.5218 × 10^−10^ (9.2651 × 10^−10^–9.6620 × 10^−10^)	Markov chain Monte Carlo
*β_g_*	Infection rate of susceptible health-care personnel in Fangcang shelter hospitals by infectious patients	3.3342 × 10^−9^ (3.3104 × 10^−9^–3.3573 × 10^−9^)	Markov chain Monte Carlo
*β_g1_*	Infection rate of susceptible health-care personnel in Fangcang shelter hospitals by asymptomatic infectious health-care personnel in Fangcang shelter hospitals	1.6957 × 10^−9^ (1.6374 × 10^−9^–1.7261 × 10^−9^)	Markov chain Monte Carlo
*γ_a_*	Recovery rate of infected asymptomatic people^d^	0.0700 (0.0691–0.0708)	Markov chain Monte Carlo
*γ*_2_	Recovery rate of infected and untreated people with symptoms (non-health-care personnel)	0.0133 (0.0131–0.0134)	Markov chain Monte Carlo
*γ*^e^	Recovery rate of patients in designated hospitals^d^	Phase I–II: 0.0089 (0.0088–0.0090) Phase III: 0.0839 (0.0829–0.0846)	Markov chain Monte Carlo
*γ_m_*	Recovery rate of patients in Fangcang shelter hospitals^d^	0.0241 (0.0238–0.0247)	Markov chain Monte Carlo
*d_w_*	Death rate from COVID-19 of infected and untreated people with symptoms (non-health-care personnel)	0.0306 (0.0304–0.0308)	Markov chain Monte Carlo
*d*^e^	Death rate from COVID-19 in designated hospitals^d^	Phase I–II: 0.0054 (0.0051–0.0056) Phase III: 0.0022 (2.1587 × 10^−3^–2.2099 × 10^−3^)	Markov chain Monte Carlo
*a*	Proportion of infected people with symptoms^d^	0.9530 (0.9502–0.9560)	Markov chain Monte Carlo
*p*	Proportion of patients admitted to designated hospitals after T_2_^f^	0.7802 (0.7763–0.7853)	Markov chain Monte Carlo
*τ*_1_	Average number of days to progress from infected to infectious, *E_w_*, *E_h_*, *E_g_*	4 (NA)	Li RY, et al.^35^
*τ*_2_	Average number of days to progress from infectious to symptomatic, *I_w1_*, *I_h1_*, *I_g1_*	3 (NA)	Li RY, et al.^35^
*k*_1_	Ratio of number of beds to health-care personnel in designated hospitals	2.486 (NA)	Wuhan Municipal Health Commission, ^22^Zhang P^23^
*k*_2_	Ratio of number of beds to health-care personnel in Fangcang shelter hospitals	1.107 (NA)	National Health Commission^24^
σ_1_	Transfer rate of patients from Fangcang shelter hospitals to designated hospitals^f^	0.02 (NA)	Bai Y^36^

CI: confidence interval; COVID-19: coronavirus disease-2019; *T*_2_: date when the first bed in the Fangcang shelter hospitals opened; *E_w_*, *E_h_*, *E_g_*: exposed people who are not health-care personnel, exposed health-care personnel who work in designated hospitals and exposed health-care personnel who work in Fangcang shelter hospitals, respectively; *I_w1_*, *I_h1_*, *I_g1_*: asymptomatic people with COVID-19 (who will develop symptoms) who are not health-care personnel, asymptomatic health-care personnel with COVID-19 (who will develop symptoms) who work in designated hospitals and asymptomatic health-care personnel with COVID-19 (who will develop symptoms) who work in Fangcang shelter hospitals, respectively; NA: not applicable.

^a^ 95% highest posterior density interval.

^b^ The infection rate of susceptible health-care personnel in designated hospitals by infectious patients in phase I of the COVID-19 epidemic is different from that in phases II and III, because of the strict measures put in place to protect health-care personnel after the first phase.^37^

^c^ Because of the strict measures put in place to protect health-care personnel, the infection rate of susceptible health-care personnel in designated hospitals from asymptomatic infectious health-care personnel in designated hospitals in phases II and III is also different from that in phase I.

^d^ Including health-care personnel.

^e^ In phase III, the recovery and death rates in designated hospitals are different from those in phases I and II because there are enough hospital beds.

^f^ Excluding health-care personnel on the assumption that health-care personnel will be given priority in use of designated hospital beds.

**Fig. 2:**
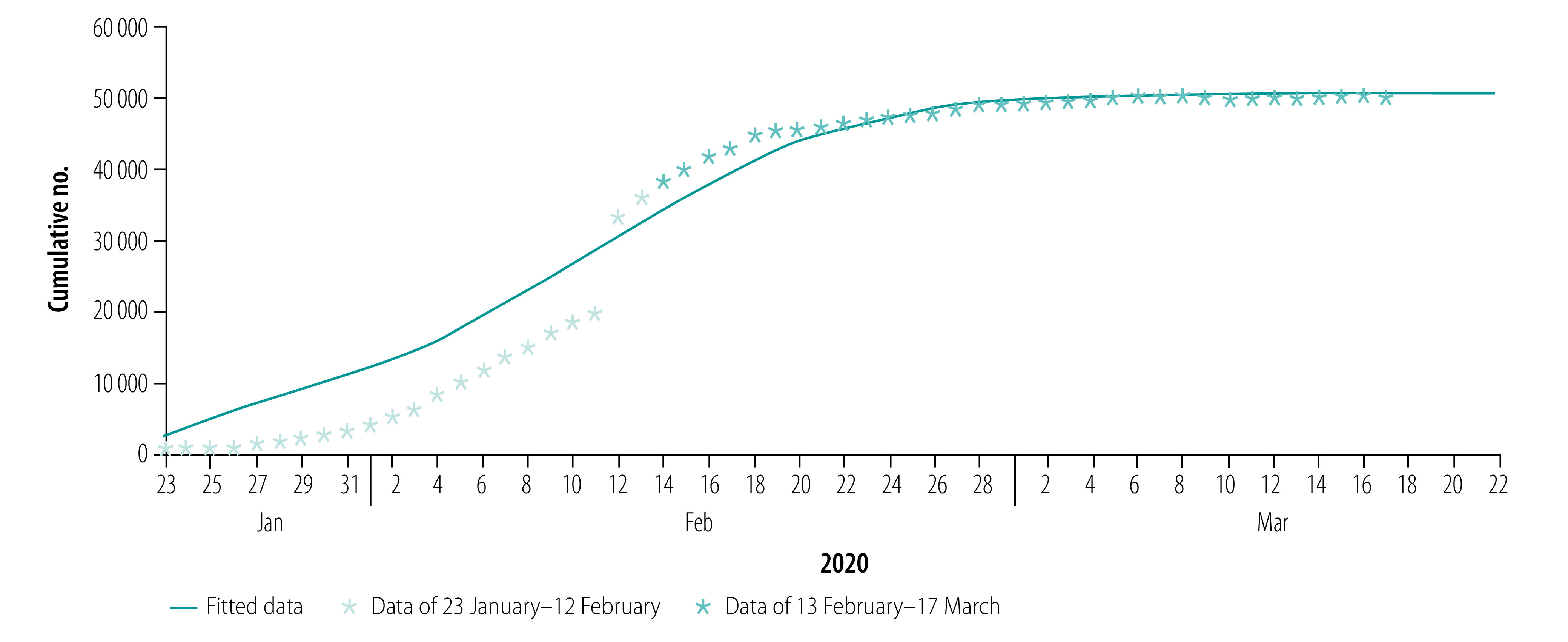
Actual and fitted data for cumulative number of COVID-19 infections, Wuhan, China, 23 January–17 March 2020

**Fig. 3:**
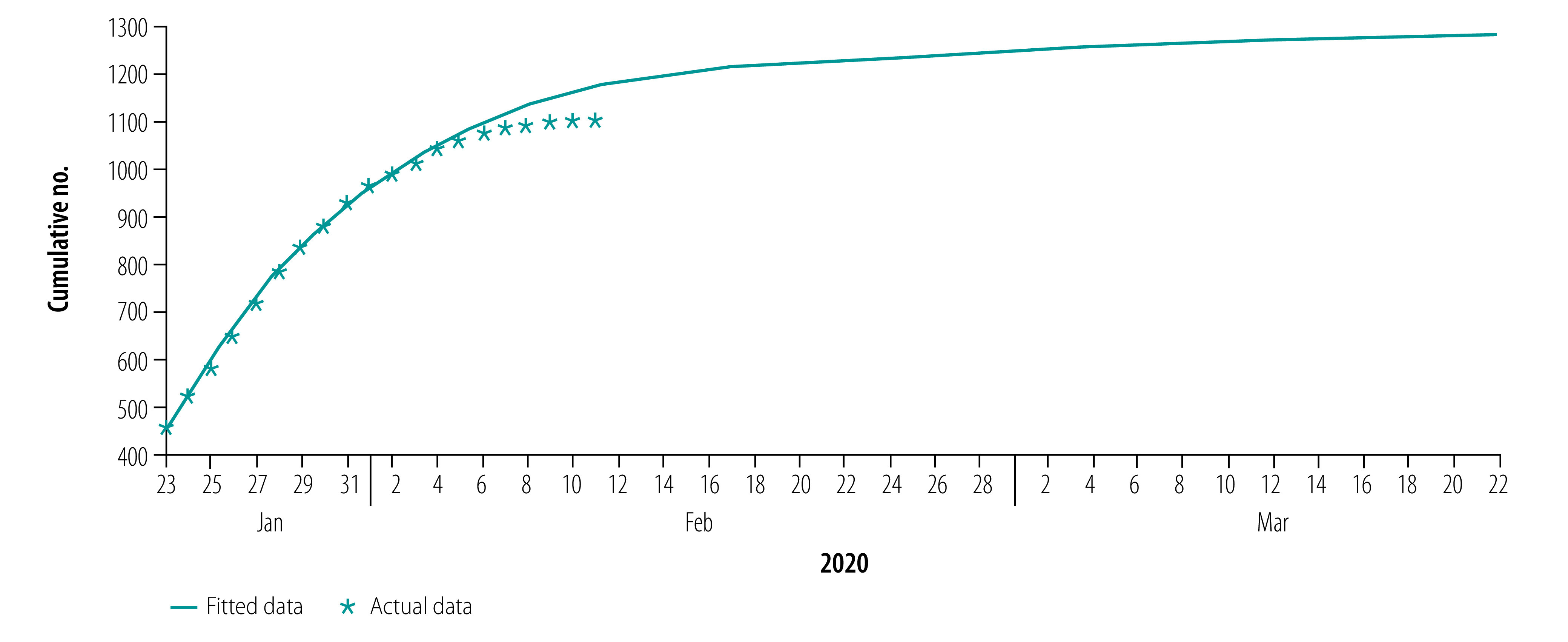
Actual and fitted data for cumulative number of COVID-19 infections in health-care personnel, Wuhan, China, 23 January–17 March 2020

**Fig. 4:**
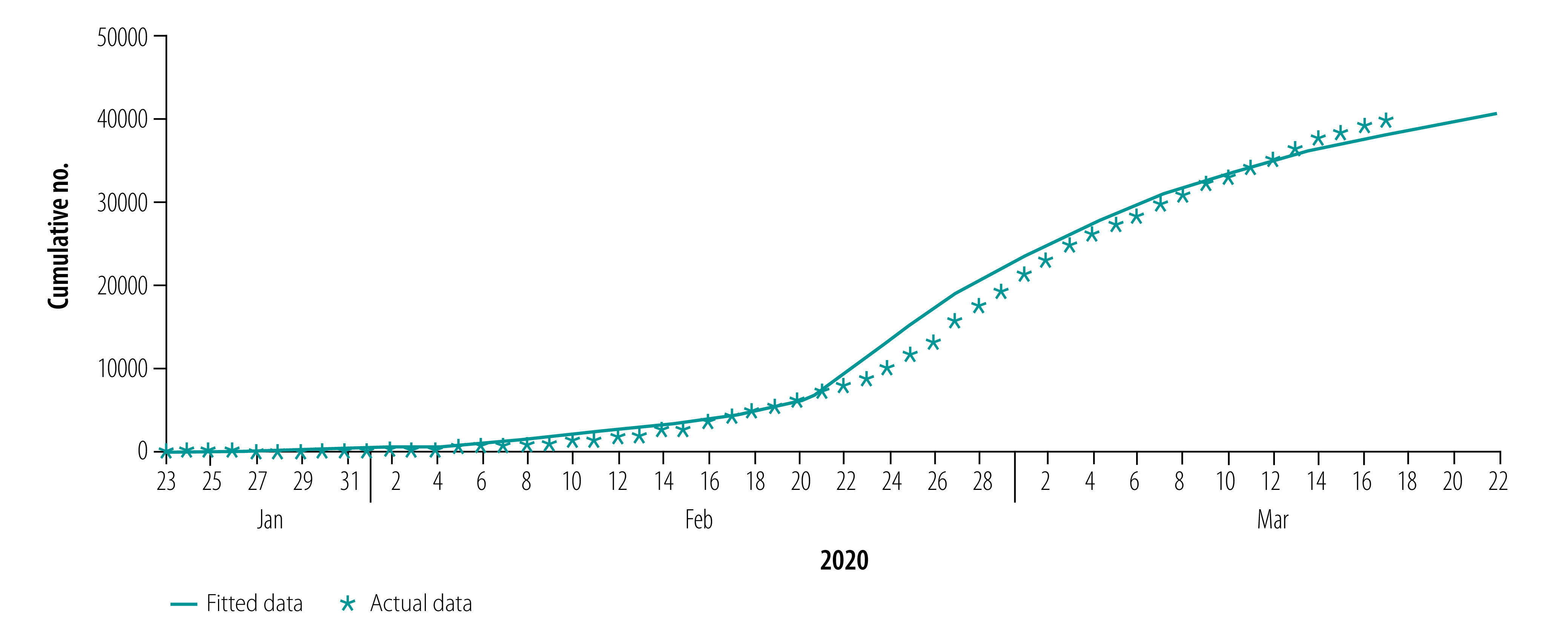
Actual and fitted data for cumulative number of recoveries from COVID-19, Wuhan, China, 23 January–17 March 2020

**Fig. 5:**
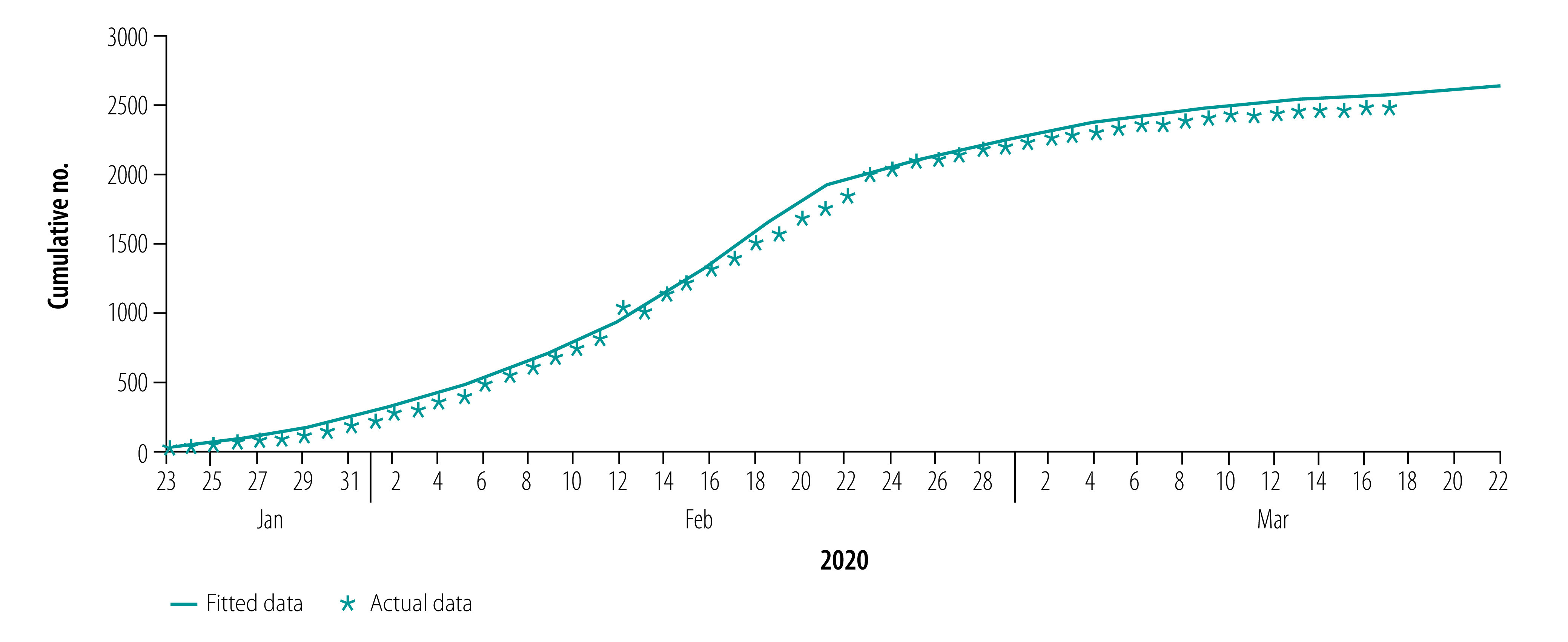
Actual and fitted data for cumulative number of COVID-19 deaths, Wuhan, China, 23 January–17 March 2020

Fig. 2, Fig. 3, Fig. 4 and Fig. 5 show our data fitting and simulation results. Our models fitted well with the normalized mean square error 0.97. We estimated that 0.95% (95% CI: 0.95–0.96) of the total exposed cases progressed to symptomatic infections (Table 2), i.e. 4.70% (95% CI: 4.40–4.98) progressed to subclinical infections, which is higher than the previously reported value of 1.20%.^13^

COVID-19: coronavirus disease-2019.

Note: The data from 23 January to 12 February (light blue star markers) were not used in the parameter estimation.

Data source: Wuhan Municipal Health Commission.^12^

COVID-19: coronavirus disease-2019.

Note: We extracted data on the cumulative number of infected health-care personnel in Wuhan, Hubei province (excluding Wuhan) and daily infected health-care personnel in the whole country^13^^,^^20^^,^^21^ to calculate the daily number of infections of health-care personnel in Wuhan from 23 January to 11 February.

COVID-19: coronavirus disease-2019.

Data source: Wuhan Municipal Health Commission.^12^

COVID-19: coronavirus disease-2019.

Data source: Wuhan Municipal Health Commission.^12^

The results of our model simulation show the peak value of the epidemic of 39 771 cases (95% CI: 39 727–39 827) on 21 February, 30 days after the lockdown on 23 January (Table 3). The total number of infections is 50 844 (95% CI: 50 757–50 915), the total number of hospital deaths is 2920 (95% CI: 2817–2985) and overall deaths (including those who died because they could not get treatment in time) is 5003 (95% CI: 4888–5065). Our model shows that the number of new cases a day falls to zero after 2 April, (95% CI: 2 April–3 April), corresponding to 71 days after implementation of the lockdown.

**Table 3:** COVID-19 epidemic size and length under different hypothetical scenarios for Fangcang shelter hospitals, Wuhan, China, 2020

Scenario	Peak size, highest no. of cases a day	Peak time, days after 23 Jan	Total no. of infections	Length of epidemic, days	Total no. of deaths	Hospital beds/1000 infected persons
**Three-phase model^a^**	39 771 (95% CI: 39 727–39 827)	30	50 844 (95% CI: 50 757–50 915)	71	5 003 (95% CI: 4 888–5 065)	3.323
Without Fangcang shelter hospitals	4 510 842	55	7 467 768	261	1 853 492	NA^b^
**Start to use Fangcang shelter hospitals**						
23 Jan	8 976	6	12 729	35	324	0.625
30 Jan	12 888	9	17 218	41	795	0.931
2 Feb	16 736	13	22 606	47	1 301	1.224
6 Feb	2 701 981	73	7 413 798	179	1 396 017	NA^b^
**No. of Fangcang shelter hospital beds**						
2*b*_2_(*t*)	21 963	16	30 164	53	1 984	1.613
1.5*b*_2_(*t*)	24 736	17	31 491	54	2 182	1.860
1.2*b*_2_(*t*)	29 592	21	37 426	60	2 566	2.282
0.8*b*_2_(*t*)	2 783 960	70	7 404 566	174	1 415 510	NA^b^
**Use of the 13 348 beds in Fangcang shelter hospitals in 1 or 2 weeks **						
1907 beds a day	28 818	19	37 892	59	2 362	2.138
953 beds a day	2 789 770	70	7 420 852	176	1 418 806	NA^b^

CI: confidence interval; COVID-19: coronavirus disease-2019; *b*_2_: number of new beds used in Fangcang shelter hospitals a day in three-phase model; NA: not applicable.

^a^ Fangcang shelter hospitals put into operation on 5 February 2020.

^b^ In this case, the number of beds cannot be used to calculate the actual ratio of hospital beds/1000 infected persons because they are too few to control the epidemic.

### Shelter hospital beds

**Fig. 6:**
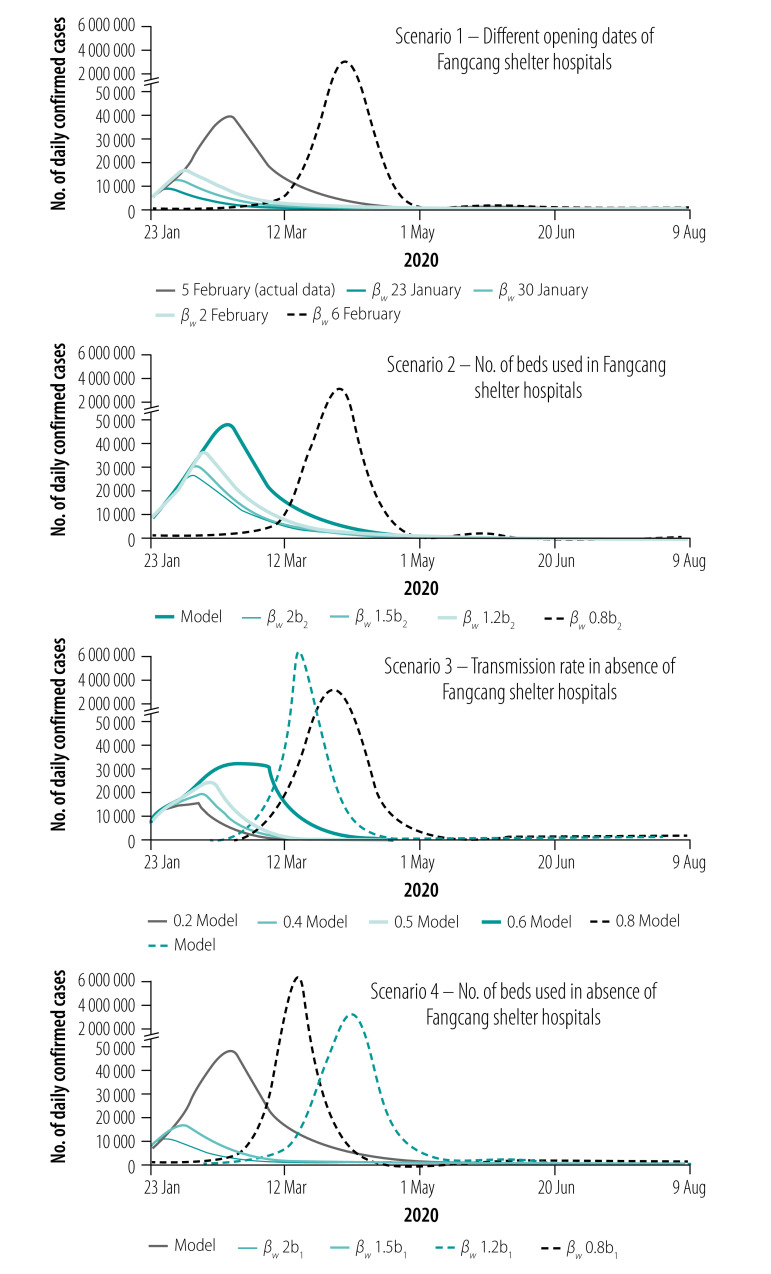
Number of daily confirmed cases of COVID-19 under different hypothetical scenarios, Wuhan, China, 23 January–9 August 2020

**Fig. 7:**
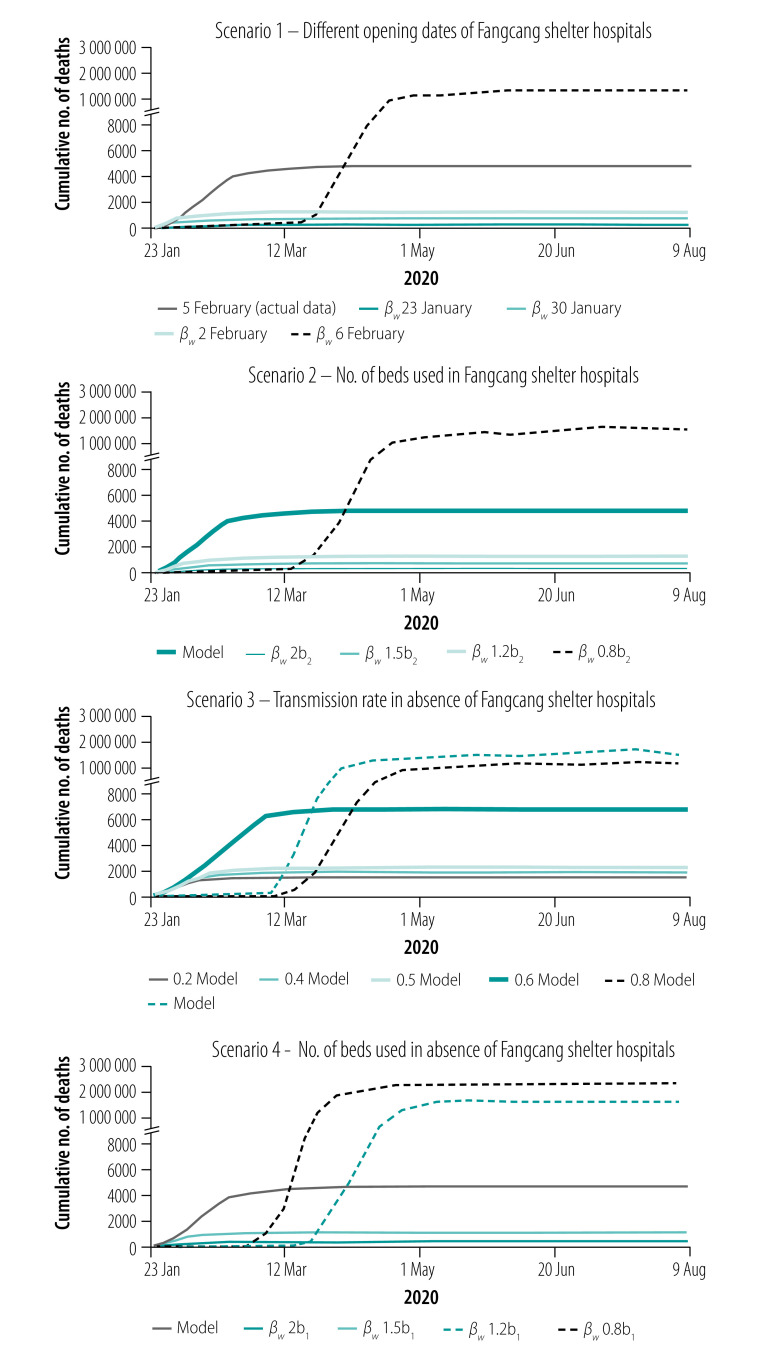
Cumulative number of deaths from COVID-19 under different hypothetical scenarios, Wuhan, China, 23 January–9 August 2020

Simulations shown in Fig. 6 and Fig. 7 (scenario 1) show the number of COVID-19 cases a day and cumulative number of deaths a day with different dates of opening the Fangcang shelter hospital beds. The models show that the earlier the Fangcang shelter hospitals open, the greater the reduction in the total number of infections, and the shorter the length of the epidemic. Our simulations show that if the Fangcang shelter hospitals opened on 30 January, the number of infections would peak on 31 January (peak value 12 888 infections), the epidemic would be shortened by 30 days, compared with the Fangcang shelter hospitals opening on 5 February, the total number of infections would decrease by 66.14% ((50 844−17 218)/50 844), and the total number of deaths would decrease by 84.11% ((5003−795)/5003; Table 3). Furthermore, in our model, if Wuhan started to build the Fangcang shelter hospitals the day the lockdown started on 23 January, the epidemic would reach its peak on 29 January with a daily peak of only 8976 infections, the total number of infections would decrease by 74.96% ((50 844−12 729)/50 844), the length of the epidemic would be 36 days shorter and the cumulative number of deaths would be reduced by 93.52% ((5003−324)/5003).

COVID-19: coronavirus disease-2019.

Notes: Scenario 1 is the number of daily confirmed cases for different opening dates of Fangcang shelter hospitals (23 or 30 January, or 2, 5 or 6 February 2020). Scenario 2 is the number of daily confirmed cases if the number of Fangcang shelter hospital beds used a day from 5 February 2020 (*b*_2_) is 1.2, 1.5 and 2 times higher than that of the model and 0.8 times lower. Scenario 3 is the number of daily confirmed cases in the absence of Fangcang shelter hospitals if the transmission rate (*β_w_*) is 0.2, 0.4, 0.5, 0.6 and 0.8 times lower than that of the model. Scenario 4 is the number of daily confirmed cases in the absence of Fangcang shelter hospitals if the number of beds used in designated hospitals is 1.2, 1.5 and 2 times higher than that of the model and 0.8 times lower.

COVID-19: coronavirus disease-2019.

Notes: Scenario 1 is the cumulative number of deaths for different opening dates of Fangcang shelter hospitals (23 or 30 January, or 2, 5 or 6 February 2020). Scenario 2 is the cumulative number of deaths if the number of Fangcang shelter hospital beds used a day from 5 February 2020 *b*_2_ is 1.2, 1.5 and 2 times higher than that of the model and 0.8 times lower. Scenario 3 is the cumulative number of deaths in the absence of Fangcang shelter hospitals if the transmission rate (*β_w_*) is 0.2, 0.4, 0.5, 0.6 and 0.8 times lower than that of the model. Scenario 4 shows cumulative number of deaths in the absence of Fangcang shelter hospitals if the number of beds used in designated hospitals is 1.2, 1.5 and 2 times higher than that of the model and 0.8 times lower. For all the scenarios with NA (not applicable), the public health system will collapse, and the original model is not applicable anymore. We simulate the case by modifying the model accordingly, considering possible herd immunity and variation of parameters for the non-healthcare group due to a lack of hospital resources.

Wuhan planned to provide 30 000 Fangcang shelter hospital beds,^27^ but by 22 February when the first empty bed became available for patients, only 13 348 Fangcang shelter hospital beds had been used. As shown in Table 3 and Fig. 6 (scenario 1), if the Fangcang shelter hospitals were launched on 6 February with 13 348 beds used, the total number of infections would reach 7 413 798 and the epidemic would last for 179 days. Therefore, if the Fangcang shelter hospitals had not opened in time to isolate the rapidly increasing number of confirmed COVID-19 cases, the epidemic would have been uncontrollable.

As shown in Fig. 6 and Fig. 7 (scenario 2) and Table 3, the total number of cases and cumulative deaths for different values of *b*_2_(*t*) (number of new beds put in use a day in Fangcang shelter hospitals) such as 1.2, 1.5 and 2 times the actual number of Fangcang hospitals beds used, the peak of the epidemic would occur 9, 13 and 14 days earlier than if *b*_2_(*t*) were unchanged, with reduced peak values of 29 592, 24 736 and 21 963, respectively. In addition, the total number of infections would be reduced by 26.39% ((50 844–37 426)/50 844), 38.06% ((50 844–31 491)/50 844) and 40.67% ((50 844–30 164)/50 844), the epidemic would last for 60, 54 and 53 days, and the cumulative deaths would decrease by 48.71% ((5003–2566)/5003), 56.39% ((5003–2182)/5003) and 60.34% ((5003–1984)/5003), respectively. However, if *b*_2_(*t*) is reduced to 0.8*b*_2_(*t*), the epidemic will not be effectively controlled (Fig. 6 and Fig. 7 (scenario 2) and Table 3). If all 13 348 beds in the Fangcang shelter hospitals were gradually used in 1 week with 1907 beds a day, our simulations indicate that the number of COVID-19 cases would peak 11 days earlier with 28 818 cases, there would be 25.47% fewer cases in total ((50 844–37 892)/50 844), the epidemic time would be shortened by 12 days, and the cumulative number of deaths would be reduced by 52.79% ((5003–2362)/5003). However, if the 13 348 beds had been opened in 2 weeks with 953 beds a day, Wuhan would have missed the opportunity of isolating the large number of confirmed cases, which would have led to over 7.4 million infections in total (Table 3).

The values of the hospital beds per 1000-infected-person ratio in different hypothetical scenarios are given in Table 3, Table 4 and Table 5. If the available hospital beds are too few to control the epidemic, it is not possible to estimate the hospital beds per 1000-infected-person ratio in the absence of additional data on the time, number of beds and method of replenishing beds in the Fangcang shelter hospitals. In general, the earlier beds in the Fangcang shelter hospitals are opened, the smaller the hospital beds per 1000-infected-person ratio needed to control the scale of the epidemic (Table 3 and Table 4). We also found that if the Fangcang shelter hospitals are not opened promptly, the hospital beds per 1000-infected-person ratio increases and does not guarantee effective control.

**Table 4:** COVID-19 epidemic size and length under different hypothetical scenarios with Fangcang shelter hospitals opened on 6 February 2020, Wuhan, China

Scenario	Peak size, highest no. of cases a day	Peak time, days after 23 Jan	Total no. of infections	Length of epidemic, days	Total no. of deaths	Hospital beds/1000 infected persons
6 Feb	2 701 981	73	7 413 798	179	1 396 017	NA
Fangcang shelter hospital 1.2*b*_2_^a^	37 497	26	48 836	68	4 290	2.976
7 Feb + 2 500^b^	33 610	23	42 282	63	3 053	2.632
9 Feb + 2 500^b^	36 468	25	46 747	67	4 064	2.893
12 Feb + 2 500^b^	41 609	28	54 116	72	4 893	3.345

COVID-19: coronavirus disease-2019; NA: not applicable.

^a^ *b*_2_ denotes number of new beds used in Fangcang shelter hospitals a day.

^b^ Number of newly added beds.

**Table 5:** COVID-19 epidemic size and length under different hypothetical scenarios if Fangcang shelter hospitals had not been used, Wuhan, China, 2020

Scenario	Peak size, highest no. of cases a day	Peak time, days after 23 Jan	Total no. of infections	Length of epidemic, days	Total no. of deaths	Hospital beds/1000 infected persons
**Three-phase models^a^**	39 771	30	50 844	71	5 003	3.323
**Without Fangcang shelter hospitals**	4 510 842	55	7 467 768	161	1 853 492	NA^b^
**Different contact infection rates^c^**						
0.2*β_w_*	12 550	16	15 026	40	1 592	1.036
0.4*β_w_*	16 094	19	19 947	46	1 992	1.277
0.5*β_w_*	19 718	22	24 679	51	2 468	1.573
0.6*β_w_*	26 344	33	37 002	68	6 954	2.197
0.8*β_w_*	2 647 611	70	7 380 173	167	1 300 171	NA^b^
*β_w_*	4 849 080	55	7 497 371	156	1 668 596	NA^b^
**Different number of designated hospital beds in use^d^**						
2*b_1_*	8 924	6	12 721	35	321	0.621
1.5*b_1_*	13 287	14	15 942	42	1 113	1.068
1.2*b_1_*	2 645 492	75	7 402 200	180	1 377 326	NA^b^
0.8*b_1_*	4 834 709	54	7 471 387	155	2 178 835	NA^b^

COVID-19: coronavirus disease-2019; NA: not applicable.

^a^ Fangcang shelter hospitals put into operation on 5 February 2020.

^b^ In this case, the number of beds cannot be used to calculate the actual ratio of hospital beds/1000 infected persons because they are too few to control the epidemic.

^c^ *β_w_* = (*β_w1_*,*β_w2_*): the combined format includes the infection rate of susceptible non-health-care personnel by asymptomatic infectious individuals who are not health-care personnel (*β_w1_*) and the infection rate of susceptible non-health-care personnel outside designated hospitals or Fangcang shelter hospitals by infectious symptomatic individuals who are not health-care personnel (*β_w2_*).

^d^ *b*_1_ is the number of new beds used in designated hospitals a day.

### Absence of shelter hospitals

Before the establishment of the Fangcang shelter hospitals, most patients were asked to quarantine or isolate at home because medical resources were insufficient. We analysed the effect of home isolation in the absence of the Fangcang shelter hospitals.

Without the Fangcang shelter hospitals, the epidemic could still be effectively controlled. Fig. 6 and Fig. 7 (scenario 3) show that medium to substantial reductions in contact transmission rates can reduce the peak number of daily infections and the final size of the epidemic, delay the peak time, and reduce the length of the epidemic. However, the total number of deaths will increase because of insufficient resources in the designated hospitals and the inability to provide immediate treatment to some critically ill patients (Table 5).

Fig. 6 and Fig. 7 (scenario 4) and Table 5 show that an increase in the capacity of designated hospitals can also effectively control the epidemic in the absence of the Fangcang shelter hospitals. When the number of designated-hospital beds is increased by 1.5 times the actual number, the peak number of daily infections is reduced to 13 287, the total number of infections is reduced to 15 942, the epidemic duration shortened to 42 days, and the total number of deaths reduced to 1113. In contrast, if the number of designated-hospital beds is reduced by 20%, the epidemic will spread on a much larger scale and last longer.

### Instantaneous risk indices 

**Fig. 8:**
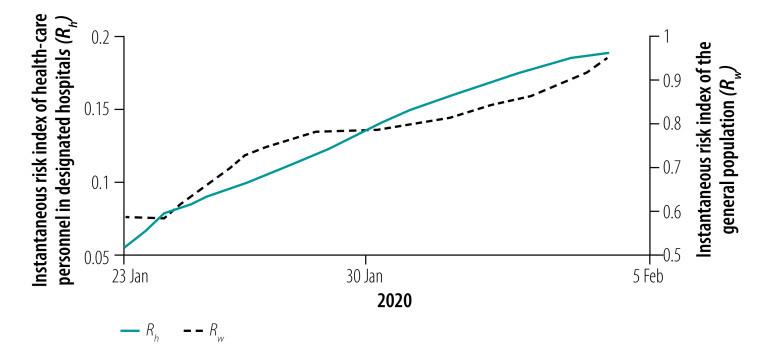
Instantaneous risk index (*R*) for the general population and health-care personnel in designated hospitals in phase I of the COVID-19 epidemic, Wuhan, China, 23 January–5 February 2020

**Fig. 9:**
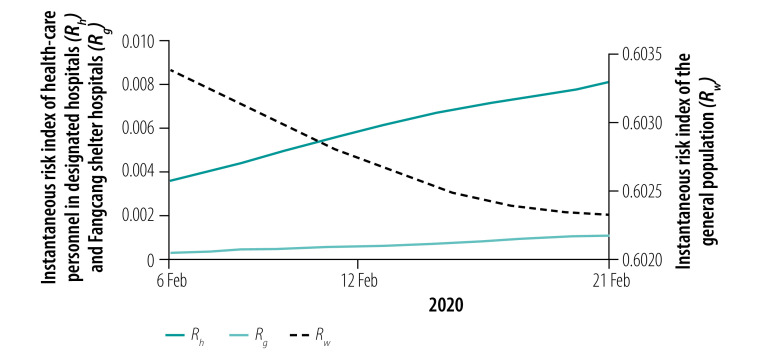
Instantaneous risk index (*R*) for the general population and health-care personnel in designated hospitals and Fangcang shelter hospitals in phase II of the COVID-19 epidemic, Wuhan, China, 6–21 February 2020

**Fig. 10:**
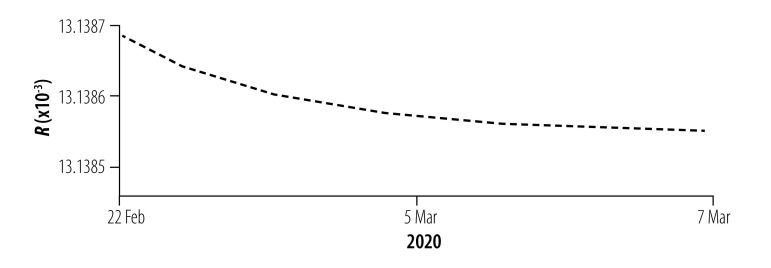
Instantaneous risk index (*R*) for the general population in phase III of the COVID-19 epidemic, Wuhan, China, 22 February–17 March 2020

**Fig. 11:**
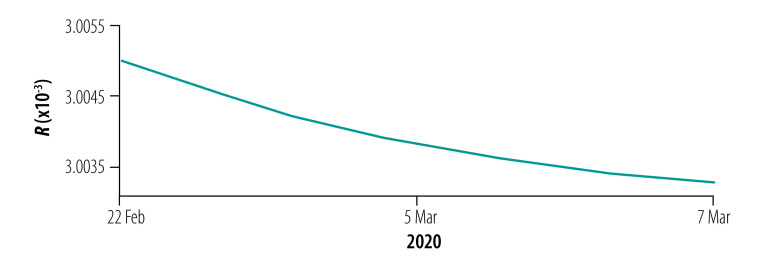
Instantaneous risk index (*R*) for health-care personnel in designated hospitals in phase III of the COVID-19 epidemic, Wuhan, China, 22 February–17 March 2020

**Fig. 12:**
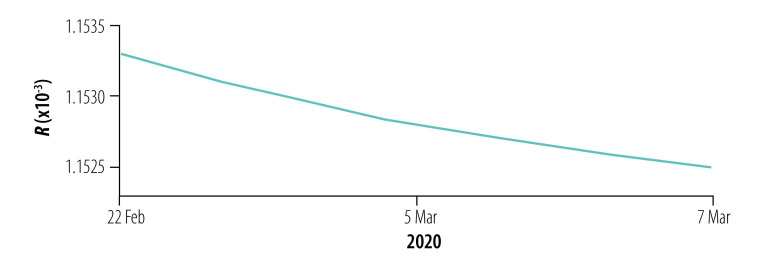
Instantaneous risk index (*R*) for health-care personnel in Fangcang shelter hospitals in phase III of the COVID-19 epidemic, Wuhan, China, 22 February–17 March 2020

We assessed the instantaneous risk index of COVID-19 in each phase of the epidemic. In phase I with the increasing number of designated-hospital beds, the risk of transmission was significantly reduced, *R*_0_(*t*) < 1, compared with the early stage of the outbreak (Fig. 8). However, the number of designated-hospital beds was not enough to cope with the increasing number of new infections, so the risk of infection to the general population, *R_w_*(*t*), was still increasing with a possibility of exceeding 1 if no more beds were added. When the Fangcang shelter hospitals were opened with a steadily increasing number of beds in phase II, *R_w_*(*t*) decreased (Fig. 9), although the risk to health-care personnel in designated hospitals and Fangcang shelter hospital, *R_h_*(*t*) and *R_g_*(*t*), respectively, continued to increase slightly until phase III when *R_w_*(*t*), *R_h_*(*t*) and *R_g_*(*t*) all decreased (Fig. 10, Fig. 11 and Fig. 12, all available at: http://www.who.int/bulletin/volumes/98/12/20-258152).

COVID-19: coronavirus disease-2019.

COVID-19: coronavirus disease-2019.

COVID-19: coronavirus disease-2019.

COVID-19: coronavirus disease-2019.

COVID-19: coronavirus disease-2019.

## Discussion

The lockdown of Wuhan provided a valuable opportunity to prevent and control the spread of SARS-CoV-2 in China and some other countries of the world.^38^ Our three-phase models mimicked and revealed how the Fangcang shelter hospitals and the group isolation policy helped to stop the epidemic in Wuhan. Our study suggests that, in lockdown cities such as Wuhan that have implemented social distancing and effective testing, if household isolation is not sufficient to inhibit the transmission of the virus, then effective group isolation of large numbers of people with mild infection in Fangcang type of facilities can curb an epidemic of COVID-19.

The success in tackling the COVID-19 epidemic in Wuhan, particularly the use of shelter hospitals, has been acknowledged and many countries have adopted a similar approach.^8^^,^^39^ For example, Italy, New Zealand and the United States of America have built temporary hospitals by transformation of caravans, ferries, trains and buses, and city squares to set up tents based on local conditions. As the pandemic has become more widespread and may last for years to come,^40^ we suggest, whenever possible and if needed, countries build more temporary hospitals such as the Fangcang shelter hospitals.

Our modelling has some limitations. Because our focus was on hospital beds and their role in mitigating and controlling the epidemic, our models are based on simplified assumptions. In addition, the data on the number of daily beds are not accurate because of counting processes and reporting. More accurate data on hospital beds will improve estimation of the parameters, but the main results of our work will not be significantly affected.

Our findings may provide policy-makers with useful information on combatting COVID-19 by considering increasing hospital-bed capacity to enhance isolation of cases where home quarantine is insufficient.
